# A Few Hints in Treating Urinary Affections

**Published:** 1852-09

**Authors:** 


*A Few Hints in treating Urinary Affections.*—Dr. Bird concludes his work on the above subject by the three following rules:—1. Whenever it is desirable to impregnate the urine with a salt, or to excite diuresis by a saline combination, it must be exhibited in solution, so diluted as to contain less than five per cent. of the remedy, or not more than twenty-five grains in an ordinary draught. The absorption of the drug into the capillaries will be ensured by a copious draught of water, or any dilutent, immediately after each dose. 2. When the urine contains purpurine, or presents other evidence of portal obstruction, the diuretics or other remedies employed should be preceded or accompanied by the administration of mild mercurials,—taraxacum, hydrochlorate of ammonia, or other cholitic remedies. By these means, or by local depletion, especially by leeches to the anus, the portal vessels will be unloaded, and a free passage obtained to the general circulation. 3. In cases of valvular disease, or other obstructions existing in the heart and large vessels, it is next to useless to endeavor to excite diuretic action, or appeal to the kidneys by remedies intended to be excreted by them. The best diuretic will, in such cases, be found in whatever tends to diminish the congested state of the vascular system, and to moderate the action of the heart, as digitalis, colchicum, and other sedatives, with mild mercurials.—*Brit. and For. Med. Chir. Rev. from the Dublin Med. Press.*

